# Supplementary material for: Support needs of south Asian adult survivors of childhood sexual abuse in the UK: Perspectives of UK mental health professionals and key stakeholders
Source: PLOS Ment Health. 2025 Oct 17;2(10):e0000454. doi: 10.1371/journal.pmen.0000454 (PMC12798236; doi:10.1371/journal.pmen.0000454)
Supplement: S1 File — (PDF) [file pmen.0000454.s001.pdf]

S1 Topic Guide for Interviews with Mental Health Professionals in UK

Initial questions about their role and work with people who have experienced trauma and PTSD/CPTSD:

1. Can you tell me a bit about your role here?
2. What kind of issues do your clients come to you with?
3. How often do you encounter clients affected by trauma?
    a. What kinds of trauma do your clients report?

Questions about working with people of South Asian origin who experienced trauma

4. How often do you see clients from South Asian origin?
    a. What are their common issues?
    b. What are the types of traumatic incidents they report?
    c. When do they bring it up?
        a. how long does it take for them to bring those events up and seek help?
    d. Would you see any difference in their issues/complaints as compared to your non-South Asian clients? (Do you notice any difference in how their lives are affected)
5. With people from South Asian origin presenting with a history of trauma, how do you approach their assessment, formulation, and treatment as compared to your non-South Asian clients?
    a. how would you try to build up a therapeutic relationship with the client?
    b. is there anything different from clients with non-South Asian background?
    c. what are some barriers in working with a trauma affected client from South Asian origin?
        i. Do they differ from your clients that are non-South Asian
    d. what are some facilitators?
        i. Do they differ?

Questions about working with people from South Asian origin who experienced abuse and/or neglect in childhood and/or occupational trauma

6. What are the difficulties reported by South Asian clients with a history of interpersonal trauma like childhood sexual abuse or neglect?
    a. What are the treatments you would offer to them
    b. how would you approach the treatment of South Asian clients with childhood sexual abuse, compared to South Asian clients with histories of child physical or emotional abuse or neglect?
7. What are the difficulties reported by South Asian clients with a history of collective trauma like disaster, war, occupational?
    a. How would you approach the treatment of South Asian clients with non-South Asian clients?
8. Could you describe any similarities or differences in the difficulties reported by South Asian CSA survivors and non-South Asian CSA survivors?

    a. How would you approach the treatment of South Asian clients with CSA, compared to non-South Asian clients with CSA?

9. From your perspective, are there any additional resources or change that are needed to better support this group (South Asian backgrounds and traumatic experiences)?
    a. specifically with childhood sexual abuse?

Is there something you would like to add which I did not ask you today?
