# Supplementary material for: Support needs of south Asian adult survivors of childhood sexual abuse in the UK: Perspectives of UK mental health professionals and key stakeholders
Source: PLOS Ment Health. 2025 Oct 17;2(10):e0000454. doi: 10.1371/journal.pmen.0000454 (PMC12798236; doi:10.1371/journal.pmen.0000454)
Supplement: Attachment — Submitted filename: V1 Response to reviewers-PLOS MH.docx [file pmen.0000454.s004.docx]

Dear Editor and Reviewers,

Thank you for your valuable feedback on our manuscript. We are very grateful for the time you have invested in carefully reviewing our work. Please see a point-by-point response to each of your comments in the table below.

Kind regards,

Authors

| **Reviewers’ Comments** | **Response to Reviewers** | **Page/section** |
| --- | --- | --- |
| Reviewer #1: I'm glad to have had the opportunity to review this paper.  The paper explores the barriers and support needs of South Asian adult survivors of childhood sexual abuse in the UK from the perspectives of mental health professionals and key stakeholders. The study is well-structured, with clear findings highlighting the cultural and systemic challenges in mental health service access. Thematic structure is logical, making the findings easy to follow.  However, while the study provides valuable insights, its sample size, lack of survivor perspectives, and limited geographic scope are significant limitations. Nevertheless, the study includes a mix of ethnic backgrounds and professional roles, strengthening its credibility.  The manuscript can be of interest upon making the following revisions. | Thank you for your feedback. |  |
| 1. Within the introduction, you may incorporate a direct comparison to prior studies on CSA survivors of other ethnic groups to highlight what makes South Asian experiences unique. | We thank the reviewer for this suggestion. We have incorporated a direct comparison between South Asian CSA survivors and survivors from Western societies. We added “As compared to adult survivors of CSA in Western societies, where individualistic cultures allow personal reflection and open expression, adult CSA survivors of South Asian origin are influenced by concepts such as izzat (honour), sharam (shame), and the pervasive culture of silence, experiencing further barriers in accessing support (Begum, 2018; Gill & Harrison, 2019).” | **Introduction, page 4** |
| 2. The introduction mentions barriers to care but does not contextualize them within existing models of cultural competency. | We thank the reviewer for this suggestion. We have added Campinha-Bacote’s model of cultural competence. We added “One way to understand the challenges faced by South Asian CSA survivors in accessing mental health support is through Campinha-Bacote’s model of cultural competence. This model outlines five interrelated aspects: cultural awareness, cultural knowledge, cultural skill, cultural encounters, and cultural desire (Campinha-Bacote, 2002). For example, the failure to address stigma or provide language-accessible services may reflect gaps in providers’ cultural knowledge and skills. Limited understanding of culture-specific values may reduce opportunities for meaningful cultural encounters.” | **Introduction, page 4** |
| 3. The rationale for qualitative methods is briefly stated but not justified. The authors may expand on why Reflexive Thematic Analysis was the most appropriate choice compared to other qualitative methods (e.g., Interpretative Phenomenological Analysis). | We thank the reviewer for this suggestion. We have expanded the justification for choice of method. We added “We chose to employ this model to draw on our professional and personal reflexivity to identify patterns, from a critical realist orientation (Braun & Clarke, 2022).” | **Methods** Data analysis, page 9 |
| 4. The purposive and snowball sampling approach is mentioned, but it is unclear how participants were screened and selected. Authors can specify inclusion/exclusion criteria (e.g., minimum years of experience, type of professional role). Furthermore, the study only includes professionals and stakeholders, with no direct survivor interviews. Authors can provide a stronger justification for this decision and discuss its implications in the limitations. | We thank the reviewer for this suggestion. We have added details on our inclusion/exclusion criteria and sampling approach in the method section and elaborated on our limitations regarding survivors’ participation. We added “Inclusion criteria for mental health professionals required participants to be qualified practitioners with at least two years of experience working with CSA survivors of South Asian origin. For key stakeholders, we required individuals to have direct experience supporting South Asian CSA survivors. ” We will be conducting a separate study interviewing survivors. We added “Future research should prioritize ethically sound approaches to include survivor perspectives, potentially through partnerships with community organisations, and using exploratory and mixed methods approaches.” | **Method:** Participants, page 8  **Discussion:** Implications, page 37 |
| 5. The paper calls for culturally adapted interventions but lacks specific action steps. Clearer and more specific implementation strategies should be provided. | We thank the reviewer for this suggestion. We have incorporated your feedback in the implications.  “In particular, this study underscores the need to adapt existing interventions to align more closely with South Asian CSA survivors’ cultural contexts. These adaptations may involve using language-appropriate materials, incorporating an understanding of family dynamics and honour-related psychoeducation, and making space for religious or spiritual beliefs in therapy. Meanwhile, interventions could be delivered through trusted community organisations or in collaboration with religious leaders to increase engagement. Culturally adapted models should be co-developed with service users and community representatives to ensure they are both effective and accessible.” | **Discussion:** Implications page 36-37 |
| 6. Please clearly state that future studies should incorporate survivor narratives to gain first-hand insights. A future mixed-methods study incorporating survivor interviews would enhance depth and applicability. | We thank the reviewer for this suggestion. We have stated the necessity to include survivor narratives and to employ exploratory and mixed methods approaches. | **Discussion:** Implications page 37 |
| 7. A thorough proofreading pass is needed before submission to check for grammatical errors. | We thank the reviewer for this suggestion. Two team members have proofread the article. |  |
| **Reviewers’ Comments** | Response to Reviewers | **Page/section** |
| Reviewer #2: Thank you for the opportunity to read such an impactful topic. I have some minor recommendations for you to address, kindly go through them:  Abstract: The abstract looks fine but I would recommend you to remove the subheadings (background and aims, method, findings, conclusion) from the abstract and make a single paragraph and concise the abstract a little more because it is exceeding the word count limit of 300 words. Check for spelling errors in findings subheadings: "Help from families and witin (within) the community as well as ...." , " This may invovle (involve) improving the ..." | Thank you for your feedback. We have removed the subheadings from the abstract and made it more concise now (286 words). We have also corrected any spelling and grammatical errors in the abstract and throughout the manuscript. | **Abstract** page 2 |
| Introduction and Discussion: Dear authors, you have wonderfully identified several barriers to access mental health services in South Asian countries in both Introduction and Discussion sections, especially for the victims of CSA, but I would encourage you to explore a little more on it. For example, one of the common barriers in of accessing Mental Health Care in South Asia is belief in superstition and easy accessibility of traditional/religious healers, village doctors/pharmacy shopkeepers. I am adding a reference for your convenience (https://doi.org/10.1007/s10597-021-00790-0) but you can explore more on this. It will help you to build strong argument, comparison and contrast in the discussion part particularly. | We thank the reviewer for this suggestion. We have added content regarding spiritual beliefs of South Asian service users, including the suggested article, both in introduction and discussion sections. We added “Mental health services across South Asia are relatively under-resourced, and individuals sometimes attribute psychological distress to supernatural causes, seeking support from religious leaders and traditional healers, due to their accessibility, affordability, and social acceptability (Subudhi et al., 2024; Koly et al., 2022).” And “Meanwhile, our study note that clients' religious and spiritual beliefs potentially influence their understanding and management of psychological distress. This observation aligns with existing literature indicating that spirituality and religious practices play a substantial role to mental health experiences in South Asian communities (Subudhi et al., 2024).” | **Introduction,** page 4  **Discussion,** page 33 |
| Again, the introduction is too long, try to concise it but, consider adding more explicit gaps in the existing literature, like, while you mention the lack of evidence, elaborating on why this gap is particularly critical for the South Asian community could strengthen the argument for the study’s significance. | We thank the reviewer for this suggestion. We have stated the necessity to fill the research gap more explicitly. We added “South Asian community’s distinct cultural norms, stigma surrounding mental health, and limited engagement with support systems can significantly shape survivors’ trauma recovery pathways (Karasz et al., 2019). Therefore, filling such research gaps is essential in informing the development of culturally responsive interventions for South Asian CSA survivors.” | **Introduction,** page 5 |
| Spelling Errors (introduction) 1st paragraph: "One of the most prevalent and debilating (debilitating) developmental...", 3rd paragraph: " Adult CSA survivors of South Asian origin often report further specific barriers in accesing (accessing) support...", 4th paragraph: "Alongside the NHS, numerous charities and organisations in the UK offer hlplines (helplines)...", 5th paragraph: "accessing community support networks, practicing cultual (cultural) healing modalities, engaging in social advocacy, and developing intentional self-care practies (practices). Studies also call for clutually-(culturally) informed interventions...", 6th paragraph, "demonstrated that evidence was availvale (available) from...", "unpleasant repsponses (responses) to...", "evidence base for specific treaments (treatments), with...", 8th paragraph: "with Saouth (South) Asian adult CSA...", "work in the USA, a crosssectional (cross-sectional) survey...", last paragraph: "UK mental health profesionnals (professionals)..." (discussion) 1st paragraph: "atment by cultually (culturally) sensitive, "limited engagment (engagement) with.." | We thank the reviewer for this suggestion. Two members have checked the spelling errors and corrected the typing mistakes throughout the manuscript. |  |
| I have gone through the rest of the manuscript where I don't have any specific recommendations. The findings are appropriate with rigorous thematic analysis. I understand the time, hard work and effort while writing a qualitative research paper, still I would highly encourage you to recheck manuscript for spelling errors too. | We thank the reviewer for this suggestion. Two team members have thoroughly proofread the manuscript now. Thank you for your wonderful feedback and valuable suggestions to improve the manuscript. |  |
