# Supplementary material for: Support needs of south Asian adult survivors of childhood sexual abuse in the UK: Perspectives of UK mental health professionals and key stakeholders
Source: PLOS Ment Health. 2025 Oct 17;2(10):e0000454. doi: 10.1371/journal.pmen.0000454 (PMC12798236; doi:10.1371/journal.pmen.0000454)
Supplement: Attachment — Submitted filename: V2 Response to the reviewers.docx [file pmen.0000454.s005.docx]

Dear Editor and Reviewers,

Thank you for your valuable feedback on our manuscript. We are very grateful for the time you have invested in carefully reviewing our work. Please see a point-by-point response to each of your comments in the table below.

Kind regards,

Authors

| **Reviewers’ Comments (Reviewer #4)** | **Response to Reviewers** | **Page/section** |
| --- | --- | --- |
| Strengths: 1. Relevance and Novelty: The study tackles an important gap in the literature by focusing on the intersection of cultural identity and CSA survivorship, particularly within the South Asian community in the UK. This is a timely and valuable contribution to mental health research.2. Qualitative Approach: The use of qualitative methods, including interviews with mental health professionals and stakeholders, is well-suited to capturing nuanced perspectives on a sensitive topic.3. Cultural Sensitivity: The manuscript demonstrates an awareness of cultural factors influencing survivors’ experiences, which is critical for informing culturally competent mental health services. | Thank you for these encouraging remarks. |  |
| Areas for Improvement: 1. - Consider refining the title to reflect the study’s scope more precisely, e.g., by specifying whether the focus is on service provision, cultural barriers, or survivor experiences as perceived by professionals  Clarity of Research Aims:- The introduction could more explicitly state the research questions or objectives. While the focus on needs is clear, specifying whether the study aims to explore barriers, service gaps, or specific support needs (or all three) would enhance clarity. | Thank you for the feedback. We have edited the title to “**Support Needs of South Asian Adult Survivors of Childhood Sexual Abuse in the UK: Perspectives of UK Mental Health Professionals and Key Stakeholders**”.  We also added a sentence in introduction section specifying our study aim: “Specifically, this study examined: barriers to help-seeking and engagement; perceived gaps in current service provision; and support needs and practical improvements to care.” | Title, Page 1  Introduction. Page 7 |
| 2. Methodology:- Participant Selection: Provide more detail on the recruitment process and inclusion criteria for mental health professionals and stakeholders. For instance, how were “key stakeholders” defined, and what expertise did they bring? This would strengthen the study’s credibility. | Thank you for the feedback. We added “defined as non-clinical roles with direct, substantive experience supporting this population (e.g., leaders or researchers within charities serving South Asian communities)” to describe how we define key stakeholders.  We have edited the “Recruitment process” providing further details on the recruitment of participants as below: “We invited professionals to take part in our study by emailing professionals in the network of JB (consultant clinical psychologist in the UK) and ST (clinical psychologist and researcher specialising in working with South Asian CSA survivors). We also posted our recruitment flyer on LinkedIn and Twitter. Additionally, we informed and invited relevant UK organisations working with adult CSA survivors and South Asian communities. Those who took part were requested to pass on the flyer to their colleagues. ” | Methodology, Page 8. |
| - Sample Size and Diversity: The manuscript would benefit from justifying the sample size and discussing the diversity of participants (e.g., professional roles, cultural backgrounds). If the sample was predominantly non-South Asian, address how this might influence perspectives on South Asian survivors’ needs. | In limitation section, we added “Although we conducted in-depth interviews, the recruited sample may not sufficiently represent the views of all UK professionals and their South Asian CSA survivors.” | Discussion, Page 36 |
| - Data Analysis: The description of the thematic analysis process is somewhat brief. Elaborate on the coding framework, whether it was inductive or deductive, and how themes were derived.  Mentioning inter-rater reliability or member-checking (if applicable) would enhance methodological rigor. | Reflexive thematic analysis developers do not endorse having a coding framework. We are grateful for your suggestion and have edited that subsection to include further details on our analysis process. For example:  “Then, YC inductively coded the data using NVivo 14 (14.24.0) to address how mental health…”  We also added “ Consistent with reflexive thematic analysis, we did not calculate inter-rater reliability; instead, we enhanced credibility via audit trail and repeated supervision meetings.” to address reliability under “quality and rigor”. | Methodology, page 10 |
| 3. Findings:- The themes presented are compelling but could be better supported with direct quotes to illustrate professionals’ and stakeholders’ perspectives. This would add depth and authenticity to the findings.   - Consider organizing the findings section to align more clearly with the research aims, ensuring each theme directly addresses the needs of South Asian CSA survivors.  Some findings (e.g., cultural stigma, lack of tailored services) align with existing literature. Clarify how your study extends or diverges from prior research to highlight its unique contribution. | Thank you for this feedback. We have included participant quotes throughout the analysis but were mindful of the length of the manuscript.  We have presented the findings based on the interaction between our themes, instead of presenting them aim-wise.  Thank you for the reminder, we have noted that some of our findings align with or are different from previous literature throughout the discussion section, for example: “ Further, our findings, in line with other studies, suggest that this ethnic group face a dual challenge of excessive stigma from within the community (Karasz et al., 2019), and limited engagement with mental health services due to disbeliefs about mental health intervention (Rastogi et al., 2014). ” |  |
| 4. Discussion and Implications:- The discussion could further explore the practical implications of your findings for mental health service providers. For example, what specific training or resources could address identified gaps? Providing actionable recommendations would enhance the study’s impact. | Thank you for the feedback. We have made a few adjustments in the implication sections including “While individual biases might be unavoidable, it would be essential to develop anti-bias programs that increase professionals’ awareness of them and minimise their impact, and incorporate such courses into the training procedure”,  “During the formulation process, it would be crucial to consider and integrate specific cultural factors such as cultural values, family dynamics and spiritual frameworks common within South Asian communities to better understand the needs of service users.” and  “These adaptations may involve using language-appropriate materials, incorporating psychoeducation plans that acknowledge cultural specific concepts, and delivering such plans in community venues, such as temples, gurdwaras, mosques, weddings, and other cultural events.” to reflect the actionable recommendation for practices. | Discussion, page 36  Discussion, page 37  Discussion, Page 27 |
| - Address potential limitations more thoroughly, such as the reliance on professionals’ perspectives rather than survivors themselves. Discuss how this choice shapes the findings and what future research could complement this approach (e.g., studies directly involving survivors). | Thank you for the feedback. We expanded our limitation by adding “Given that many South Asian CSA survivors face significant obstacles to seeking support, professionals’ accounts inevitably capture the limited challenges and needs that are visible to services, while more hidden lived experiences remain underrepresented. ” | Discussion, page 35 |
| - The manuscript could benefit from a brief discussion of how findings might apply to other minority ethnic groups or contexts, broadening its relevance. | Thank you for the feedback, we added “Finally, several mechanisms that this study identified, such as stigma, interpreter-mediated confidentiality, cultural framing of emotion, may generalise across other minority communities, provided that appropriate regulations and contextual considerations are taken into account (Knifton, 2012). ” to address the potential transferability of our findings. | Discussion, page 35 |
| 5. Ethical Considerations:- While ethical approval is mentioned, expand on how sensitive topics were handled during interviews (e.g., ensuring participant wellbeing, managing potential distress). This is particularly important given the study’s focus on a vulnerable population. | We added “Before interviews, participants were informed about the sensitive nature of CSA discussions; they were then reminded of their right to pause, skip, or stop the conversation without consequences. We also included details on data collection, storage and contact means of researchers in our information sheet. ” in data collection section. | Methodology, page 9 |
| 6. Clarity and Structure:- Some sections, particularly the results, could be more concisely written to avoid repetition. For example, streamline overlapping themes or sub-themes to improve readability.- Ensure consistent terminology (e.g., “South Asian survivors” vs. “adult survivors of CSA”) throughout the manuscript for clarity.- A table summarizing key themes and sub-themes could help readers quickly grasp the study’s findings. | Thank you for the feedback, we standardised term to “South Asian CSA survivors” after first definition. We also checked the writing to improve readability.  We already have a table stating the themes and subthemes but their summaries may not fit neatly within that table. |  |
| Additional Suggestions:- Consider integrating a brief literature review on existing studies about CSA survivors in minority communities to contextualize your findings within a broader framework.  - If possible, include a section on how the findings were disseminated or could be shared with relevant stakeholders (e.g., mental health organizations, South Asian community groups) to maximize real-world impact. | Thank you for these additional suggestions. We would want to keep the focus on South Asian adult CSA survivors as there is limited literature on them. We are developing this work with these adult survivors of South Asian origin in the UK. There is a scoping review and a focus group study underway.  Thank you for the helpful advice on dissemination. We sent the summary of findings to our participants. As a next step, the last author has conducted a scoping review and qualitative study (in prep) with professionals in the UK. Based on this present manuscript and the review and new qualitative study, we will be working with organisations to improve the resources and access to services for South Asian survivors in the UK. |  |
| **Reviewers’ Comments (Reviewer #5)** | **Response to Reviewers** | Page/section |
| It is an interesting, unique study giving insight into the “Needs of South Asian Adult Survivors of Childhood Sexual Abuse in the UK: A Qualitative Study with UK Mental Health Professionals and Key Stakeholders.” However, here are some comments for your attention | Thank you for these encouraging remarks. |  |
| Abstract: The abstract is clear, however; Is it possible to adapt the aim of the study that is captured in the last paragraph of the introduction, “to explore the perspectives of UK mental health professionals and key stakeholders on the treatment and support needs of South Asian adult CSA survivors,” for uniformity purposes? | Thank you for the feedback, we have revised the abstract and edited “This study aimed to explore the perspectives of UK mental health professionals and key stakeholders on the treatment and support needs of South Asian adult CSA survivors. ” | Abstract, page 2 |
| Introduction: The introduction lacks a clear definition of childhood sexual abuse (CSA), including how CSA presents in the survivors.  Could you include some statistics (prevalence) on childhood sexual abuse (CSA) survivors in the UK? | Thank you for the feedback. We added the definition of CSA “One of the most prevalent and debilitating developmental traumas, childhood sexual abuse (CSA), defined as sexual activity involving a child who is incapable of giving consent, enacted through power, coercion, or exploitation (Mathews and Collin-Vézina, 2019; Russell et al., 2020).”  We also added prevalence statistics on CSA “ In the UK, the Crime Survey for England and Wales estimated that 7.5% of adults aged 18-74 years experienced CSA before age 16, underscoring the public-health significance of tailored support (ONS, 2020). ” | Introduction, Page 3  Introduction, page 3 |
| Methods: Under the sub-heading of data collection, use “interview guide” instead of “topic guide.”  Was the interview guide pretested after its development, and where was it pretested?  How did you come up with a sample size of five mental health workers and two stakeholders? Was this it after reaching redundancy? | Thank you for the feedback. Term “topic guide” was replaced by “interview guide”.  We explained the pretest of interview guide “The interview guide for this study was adapted from a guide developed by ST, RA and JB, and previously used in a related qualitative study led by ST with mental health professionals and key stakeholders in South Asia (Talwar et al., 2024), YC and ED further revised and contextualised the interview guide for UK services. YC, after conducting the first two interviews, edited the topic guide further in response to the completed interviews, and in consultation with ST.”  Regarding sample size, we did not use data saturation, as saturation is not a necessary criterion in reflexive thematic analysis. We focused on inviting diverse views. | Methodology, Page 9 |
| Results: Is it possible to include more details in “Table 1: Characteristics of participants,” such as duration of practice, as this may go hand in hand with experience as regards offering treatment to CSA survivors? | Thank you for the feedback. We did not systematically collect years-in-practice; however, inclusion criteria required at least 2 years post-qualification for mental health professionals. |  |
| Discussion: In the first paragraph of the discussion, the statement “We also found that there may be a need to better understand the needs of South Asian CSA survivors in the UK” is confusing, as the whole study is about this. I suggest that this can be removed.  Regarding reflexivity, the authors elaborated it in the methodology section; however, during the discussion of the themes (findings), they did not acknowledge/incorporate their positionality in the narration of the themes. A reflexivity statement is key at the end of each of the broad themes discussed. | Thank you for the feedback, we have removed the quoted sentence.  Regarding reflexivity, we discussed our backgrounds and positioning under “reflexivity” and explain how our professional and personal backgrounds informed the interpretation of all themes. At the end of the themes, having a reflexivity statement may not have been any different that the one in the methods section as it detailed our positioning throughout the study, including analysis. |  |
| **Reviewers’ Comments (Reviewer #6** **)** | **Response to Reviewers** | Page/section |
| General comments and some key concerns: 1. The manuscript is addressing an important topic on the “Needs of South Asian Adult Survivors of Childhood Sexual Abuse in the UK: A Qualitative Study with UK Mental Health Professionals and Key Stakeholders”. The barriers and support needs of South Asian adult survivors of childhood sexual abuse in the UK is critical in the perspectives of the of mental health professionals and key stakeholders. However, there some comments that needs to be addressed including the following: | We appreciate these encouraging remarks. |  |
| Language: There is excessive use of first and second person in sentence construction and suggest that they are reduced in the abstract and all through the manuscript. They should be used only where necessary.  Note: The authors need to align all the components of the study on the perspectives of the UK Mental Health Professionals and Key Stakeholders on the needs of South Asian Adult Survivors of Childhood Sexual Abuse in the UK since the South Asian Adult Survivors of CSA did not participate in the study. | Thank you for the feedback. We chose to write in first person active voice in line with the APA writing style recommendations.  We also clarified the perspectives of professionals instead of survivors throughout the manuscript. |  |
| Title: The authors need to paraphrase the title to reflect the actual study participants in the study since the actual South Asian Adult Survivors of Childhood Sexual Abuse (CSA) did not participant in the study. Suggestion “Needs of South Asian Adult Survivors of Childhood Sexual Abuse in the UK: Perspective from UK Mental Health Professionals and Key Stakeholders”. The title and the objective need to be paraphrased to specifically reflect on the actual study participants. | Thank you for the feedback, we have changed the title to address the “Perspective of” professionals and stakeholders. | Title, Page 1 |
| 2. Abstract: The first sentence on Childhood sexual abuse (CSA), the authors should provide information where this problem is as a background to the study; and suggest UK where the study was conducted (see the title). The authors need to address the Childhood sexual abuse (CSA) globally and then narrow down to the UK in both the abstract and introduction. The aim of the study in the abstract is not in line with the title and therefore it needs to be paraphrased.   The authors should clearly explain who are the 7 study participants and 2 stakeholders. Were the adult survivors of CSA of South Asian origin, the study participants? It is not clear.  Was there any tool that was used in the interviews? The authors should provide the key finding from the study and the conclusion should be based on those findings.  The authors need to paraphrase the objective to specifically reflect on the actual study participants in the study. | Thank you for your feedback. We clarified the UK aspect in the abstract “ In the UK, South Asian CSA survivors may face distinctive cultural and structural barriers to care.”  We have stated in the abstract clearly that we conducted interviews with mental health professionals and key stakeholders.  We have included that we used an interview guide to conduct the interviews as below:  “We conducted semi-structured, in-depth interviews using interview guides with mental health professionals and key stakeholders working with adult CSA survivors and other trauma survivors of South Asian origin in the UK.”  We have edited the abstract further to highlight the key findings and conclusions. We rephrased the aim “This study aimed to explore the perspectives of UK mental health professionals and key stakeholders on the treatment and support needs of South Asian adult CSA survivors. ” | Abstract, Page 2  Abstract, page 2  Methodology, page 9  Abstract, page 2 |
| 3. Introduction: The authors should stick to childhood sexual abuse (CSA) and not global trauma, and this should highlight the CSA problem globally and then narrow down to the South Asian Adult Survivors of Childhood Sexual Abuse in the UK and the development of mental illnesses especially the Post-Traumatic Stress Disorder (PTSD). The authors should also explain the need of the diverse CSA globally both developed and under-developed including the South Asia that have been reported which possibly may differ from those of the South Asian origin i.e. do CSA in South Asia, sub- Saharan Africa and western Europe and many others differ or share similarity in this aspect. This should be highlighted in a holistic way and not only the South Asia since they have different cultural diversity and resilience.   The authors should clearly explain the knowledge gap the study tried to address. Then the aim of the study should be in line with the title. | Thank you for your feedback. We emphasized the focus of CSA in our study by adding a definition and prevalence “One of the most prevalent and debilitating developmental traumas, childhood sexual abuse (CSA), defined as sexual activity involving a child who is incapable of giving consent, enacted through power, coercion, or exploitation ”. “ In the UK, the Crime Survey for England and Wales estimated that 7.5% of adults aged 18-74 years experienced CSA before age 16, underscoring the public-health significance of tailored support (ONS, 2020). ”  We also added “Specifically, this study examined: barriers to help-seeking and engagement; perceived gaps in current service provision; and support needs and practical improvements to care. ”to readdress the aim of this study. | Introduction, Page 3  Introduction, Page 7 |
| 4. Methods section: The authors should replace “Methods” with “Methodology”.   The authors should clearly state the study setting and the study participants. Who were the actual adult CSA survivors?  The authors should also explain why the CSA were not included in the study so that their needs perspectives are known other than getting them from the professionals.  For the key stakeholders, they wouldn’t be individuals with experience supporting South Asian CSA survivors; they should be the actual CSA survivors.  The authors should explain how snowball sampling was conducted and who was used to identify those individuals of interest.  The sample size seems to be small for the generalization of the finding and can the authors explain why this was so?  Was there a tool that was used as a guide during the intervention?  For how long were the interviews for each study participants and when were they conducted? This information need to be incorporated in each relevant sub-section.  On the subsection of quality and rigor, the authors recruited participants from diverse backgrounds, including ethnicity, gender, age, and occupation, to capture a range of opinions, these should be clearly explained.  And given this criteria, how many participants represented each of these criterion (takes back to the sample size and generalization of the findings).  On the Reflexivity, the authors mention that all team members, how many teams, and the numbers in each team or individuals that were making each team. | Thank you for the feedback. Title “Methods” was replaced with “Methodology”.  We have made clear that “No survivors took part in this study.”  For the present study, we wanted to understand the perspectives of professionals only. Therefore, we did not invite adult CSA survivors. We have studies underway, including exploring survivors’ views.  By key stakeholders, we mean voluntary sector professionals supporting survivors. We agree one of the key stakeholders would be survivors. We have clarified this by defining key stakeholders in methodology: “For key stakeholders, defined as non-clinical roles with direct, substantive experience supporting this population (e.g., leaders or researchers within charities serving South Asian communities)”.  In snowball sampling, those professionals who gave consent to take part and attended the interviews were requested to simply pass on our study information to their colleagues. We have added these details in methods as below: “We invited professionals to take part in our study by emailing professionals in the network of JB (consultant clinical psychologist in the UK) and ST (clinical psychologist and researcher specialising in working with South Asian CSA survivors).”  Since this was a qualitative study, our focus was on transferability of findings by having a diverse team and participants, with representation from South Asian backgrounds. Our personal backgrounds and professional clinical and qualitative research experiences were helpful in designing the study and the interview guide. We have added further details under ‘quality and rigor’. However, we noted our small sample size as a limitation in the discussion section and proposed future study direction.  We developed interview questions to lead the interviews. We added description to our interview guide in methodology section “The interview guide for this study was adapted from a guide developed by ST, RA and JB, and previously used in a related qualitative study led by ST with mental health professionals and key stakeholders in South Asia (Talwar et al., 2024), YC and ED further revised and contextualised the interview guide for UK services. YC, after conducting the first two interviews, edited the topic guide further in response to the completed interviews, and in consultation with ST.”  Details of our participants were addressed in table 1, average duration of the interviews was noted in results as well “The interviews lasted between 30 minutes 26 seconds and 59 minutes 53 seconds, with an average duration of 39 minutes 40 seconds.”  In quality and rigorous, we have mentioned that we aimed to recruit a diverse sample: “The study aimed to recruit participants from diverse backgrounds, including ethnicity, gender, age, and occupation, to capture a range of opinions.” In results, we have highlighted the ethnic and experience based diversity: “ Six participants identified as female, and one as male. In terms of ethnicity, one participant was Black British, two were British Indian, and four participants were White (British, German and Irish). The age of our participants ranged between 30 to 66 years, with a mean age of 47 years. ”. In Strength, we have highlighted the diversity of participants: “Fourth, our participants were from diverse ethnicities, providing a more comprehensive understanding of the subject.”  All our participants were recruited only after meeting our eligibility criteria. We have given details of their characteristics in Table 1.  There is only one research team for this study, all members’ background was listed in reflection section. | Methodology, page 7  Methodology, Page 8  Methodology, page 8  Methodology, page 8  Methodology, page 9 |
| 5. Results: The study explored the needs of South Asian Adult Survivors of Childhood Sexual Abuse in the UK, the lack of the representation of South Asian Adult Survivors of CSA as the title and aim suggests limits the key findings on this study group. Furthermore, only 2 British Indian (Table 1) who were not CSA represented the CSA group with lack of that experience is limitive for the generalization of the CSA experiences. The title and the objective need to be paraphrased to specifically reflect on the actual study participants.   The findings should be presented as % (n) in table 1 and to one decimal place.  The mean age should have a standard deviation.  In table 2, the finding on the challenges faced by survivors with professionals should be written as “challenges faced by survivors reported by professionals” since the survivors did not participate in the study.  The authors should explain the common mental disorders encountered by the South Asian Adult Survivors of Childhood Sexual Abuse in the UK that is reported and this should also appear in the discussion. | Thank you for the feedback. We have addressed the lack of survivors’ representation, as well as the sample size as limitation, and discussed the future direction.  We have added percentage in table 1 and to two decimal place.  Including a standard deviation for this sample size may not add much value.  The finding “challenges faced by survivors with professionals ” emphasized professional-reported survivors experiences during the process of interacting with professionals, we re-addressed “In this study, participants reported that their South Asian adult CSA survivor clients, at times, faced difficulties during the interaction with the professionals.”  We added in our discussion: “Across interviews, professionals most frequently described their clients’ symptoms consistent with PTSD/CPTSD, alongside depressive and anxiety symptoms. ” | Result, Page 23  Discussion, page 33 |
| 6. Discussion: The authors should explain the common mental disorders encountered by the South Asian Adult Survivors of Childhood Sexual Abuse in the UK that is reported and how this is related previous studies elsewhere.   Among the limitations of the study mentioned, the small sample size on each criterion including the diverse backgrounds, including ethnicity, gender, age, and occupation, to capture a range of opinions should be included.  On the implications for policy, the challenge of the small sample size and the lack of the lived examples in the study limit the policy development and the intervention development towards the South Asian Adult Survivors of Childhood Sexual Abuse in the UK since findings are based on the individual opinions of the healthcare professionals. | In discussion, we added “Across interviews, professionals most frequently described their clients’ symptoms consistent with PTSD/CPTSD, alongside depressive and anxiety symptoms. These patterns mirror international evidence on adult CSA survivors in non-UK settings. ”  In limitation, we added “Given that many South Asian CSA survivors face significant obstacles to seeking support, professionals’ accounts inevitably capture the limited challenges and needs that are visible to services, while more hidden lived experiences remain underrepresented. ” to address the potential influence of limited perspectives provided on professionals and stakeholders solely.  We agree, this was an exploratory study. Our implications are based on this small yet diverse sample. Therefore, it has important implications for policy, which we have tentatively stated, considering the number of participants. However, this is the first study to explore professionals’ views on services for adult CSA survivors of South Asian origin in the UK, laying the foundation for future work. | Discussion, page 33  Discussion, page 35 |
| 7. Conclusion: The authors should clearly provide the key finding from the study especially on the common mental disorders encountered by the CSA as reported by the health professionals and the key challenges encountered by the CSA in UK. | We added “Overall, mental health professionals reported frequent observation of post-traumatic symptoms, depression, and anxiety, and highlighted barriers specific to the survivors’ circumstances and those related to professionals.” | Conclusion, Page 38 |
